# TREM2 Promotes Immune Evasion by Mycobacterium tuberculosis in Human Macrophages

**DOI:** 10.1128/mbio.01456-22

**Published:** 2022-08-04

**Authors:** Ankita Dabla, Yi Chu Liang, Nusrah Rajabalee, Courtney Irwin, Carolyn G. J. Moonen, Jessie V. Willis, Stefania Berton, Jim Sun

**Affiliations:** a Department of Biochemistry, Microbiology and Immunology, University of Ottawa, Ottawa, Ontario, Canada; b Centre for Infection, Immunity and Inflammation, University of Ottawa, Ottawa, Ontario, Canada; Max Planck Institute for Infection Biology

**Keywords:** *Mycobacterium tuberculosis*, triggering receptor expressed on myeloid cells, TREM2, macrophage cell death, type I interferon, IFN, reactive oxygen species, phagocytosis

## Abstract

Macrophage surface receptors are critical for pathogen defense, as they are the gatekeepers for pathogen entry and sensing, which trigger robust immune responses. TREM2 (triggering receptor expressed on myeloid cells 2) is a transmembrane surface receptor that mediates anti-inflammatory immune signaling. A recent study showed that TREM2 is a receptor for mycolic acids in the mycobacterial cell wall and inhibits macrophage activation. However, the underlying functional mechanism of how TREM2 regulates the macrophage antimycobacterial response remains unclear. Here, we show that Mycobacterium tuberculosis, the causative agent for tuberculosis, specifically binds to human TREM2 to disable the macrophage antibacterial response. Live but not killed mycobacteria specifically trigger the upregulation of TREM2 during macrophage infection through a mechanism dependent on STING (the stimulator of interferon genes). TREM2 facilitated uptake of *M. tuberculosis* into macrophages and is responsible for blocking the production of tumor necrosis factor alpha (TNF-α), interleukin-1β (IL-1β), and reactive oxygen species (ROS), while enhancing the production of interferon-β (IFN-β) and IL-10. TREM2-mediated blockade of ROS production promoted the survival of *M. tuberculosis* within infected macrophages. Consistent with this, genetic deletion or antibody-mediated neutralization of TREM2 reduced the intracellular survival of *M. tuberculosis* through enhanced production of ROS. Importantly, inhibition of type I IFN signaling in TREM2-overexpressing macrophages restored the ability of these cells to produce inflammatory cytokines and ROS, resulting in normal levels of intracellular bacteria killing. Collectively, our study identifies TREM2 as an attractive host receptor for host-directed antimycobacterial therapeutics.

## INTRODUCTION

Despite its existence for centuries, tuberculosis (TB) remains one of the leading causes of infectious disease-related deaths, causing over 1.5 million deaths and 10 million new active cases globally each year (1). The causative agent of TB is the airborne bacterial pathogen Mycobacterium tuberculosis, which has coevolved with humans to establish a highly successful continuous cycle of inhalation, active-to-chronic infection, latency, dissemination, and transmission to other individuals to ensure its replication and persistence.

Macrophages are the primary host cell for *M. tuberculosis* and represent the first line of immune defense against respiratory pathogens (2). Macrophages sense mycobacterial pathogen-associated molecular patterns (PAMPs) that are secreted or present on the *M. tuberculosis* surface via a variety of surface receptors, such as Toll-like receptors (TLRs), C-type lectin receptors (CLRs), and scavenging receptors (3). Sensing of *M. tuberculosis* by these receptors results in activation of signaling cascades that drive the production of multiple proinflammatory cytokines and promote antigen presentation, as well as launching antimycobacterial mechanisms, such as production of reactive nitrogen intermediates (RNI) and reactive oxygen species (ROS), phagolysosome fusion and acidification (4), and autophagy (5). In addition to acting as PAMPs, mycobacterial cell wall components can also act as bacterial effector molecules, and *M. tuberculosis* has evolved strategies to utilize these PAMPs to exploit or preferentially target host receptors in order to manipulate immune responses and facilitate its intracellular survival, replication, and persistence (3, 6). *M. tuberculosis* produces specific proteins, such as EsxA (Rv3875) and Hsp60 (Rv0440), that bind to TLRs to skew the balance between pro- and anti-inflammatory cytokine production (7, 8). *M. tuberculosis* can also evade TLR activation via surface phthiocerol dimycocerosates (PDIMs) that mask its PAMPs from TLR recognition (9). CLRs, such as mannose receptors (MR), DC-SIGN, and Mincle, are also exploited by *M. tuberculosis* to create a more amenable environment. Binding of mannose-capped lipoarabinomannan (manLAM) and trehalose-6,6′ dimycolate (TDM) on the *M. tuberculosis* cell wall to MR and Mincle, respectively, inhibits inflammatory responses to promote *M. tuberculosis* survival (10–12). Host-protective sensing and internalization of *M. tuberculosis* trigger generation of ROS via the activation of NADPH oxidase (NOX2) (13), which produces highly reactive intermediates that are capable of destroying bacterial membrane lipids, proteins, and DNA through oxidation. However, *M. tuberculosis* is able to evade this key innate immune response by neutralizing radicals or inhibiting the assembly of NOX2 in the phagosome membrane (14, 15) or by bypassing specific receptors (16). Macrophages also produce type I interferons (IFNs) in response to *M. tuberculosis* infection (17, 18), which promote disease progression by inhibiting the production of important cytokines, such as interleukin-2 (IL-12) and tumor necrosis factor alpha (TNF-α), and limit ROS production to promote the intracellular survival of *M. tuberculosis* (19, 20).

TREM2 (triggering receptor expressed on myeloid cells 2) is a surface receptor that belongs to the family of TREM transmembrane glycoproteins. It was first identified in 2000 and is widely distributed on the surface of immune cells, such as macrophages, microglia, dendritic cells, and osteoclast precursors (21). The TREM2 receptor is composed of an Ig-like V-type extracellular domain, a transmembrane region, and a cytoplasmic tail. In the cytoplasmic domain, TREM2 associates with the adaptor molecule DAP12 (DNAX activating protein 12), which possesses an immunoreceptor tyrosine-based activation motif (ITAM) to regulate cell functions (21, 22). TREM2 is well characterized in neurodegenerative diseases (23) and has been shown to mediate diverse functions in microglia, such as phagocytosis (24), inflammatory signaling (25, 26), and proliferation and survival (27). A role for TREM2 in mediating inflammation and activation of macrophages has also emerged for various bacterial, viral, and parasitic infections (28–30). In the context of mycobacterial infections, Lizasa et al. recently showed that *M. tuberculosis* and Mycobacterium bovis BCG bind to murine TREM2 via mycolic acid-containing lipids on its cell wall (31). Importantly, their study indicated that TREM2 contributed to mycobacterial evasion of host immunity by limiting the activation of mycobactericidal macrophages. However, key conclusions from that study were based on the use of lipid ligands or *M. bovis* BCG. As such, the functional role and mechanism of TREM2 during *M. tuberculosis* infection in human macrophages remain unclear.

In the present study, we demonstrate that *M. tuberculosis* binds to human TREM2 and that infection induces an upregulation of this receptor in human macrophages through a mechanism dependent on STING (the stimulator of interferon genes). Accordingly, elevated levels of TREM2 increased uptake of *M. tuberculosis*, but rendered human macrophages more permissible to *M. tuberculosis* infection through a type I IFN-driven mechanism that reduced production of ROS. Additionally, we found that deletion or inhibition of TREM2, or blockade of type I IFNs, promoted production of inflammatory cytokines, ROS, and cell death, leading to decreased intracellular survival of *M. tuberculosis*. These results suggest that *M. tuberculosis* exploits the anti-inflammatory functions of TREM2 to evade host immunity, which may have important implications in the development of host-directed treatments for TB.

## RESULTS

### Human TREM2 binds to *M. tuberculosis* and contributes to phagocytosis.

**FIG 1:**
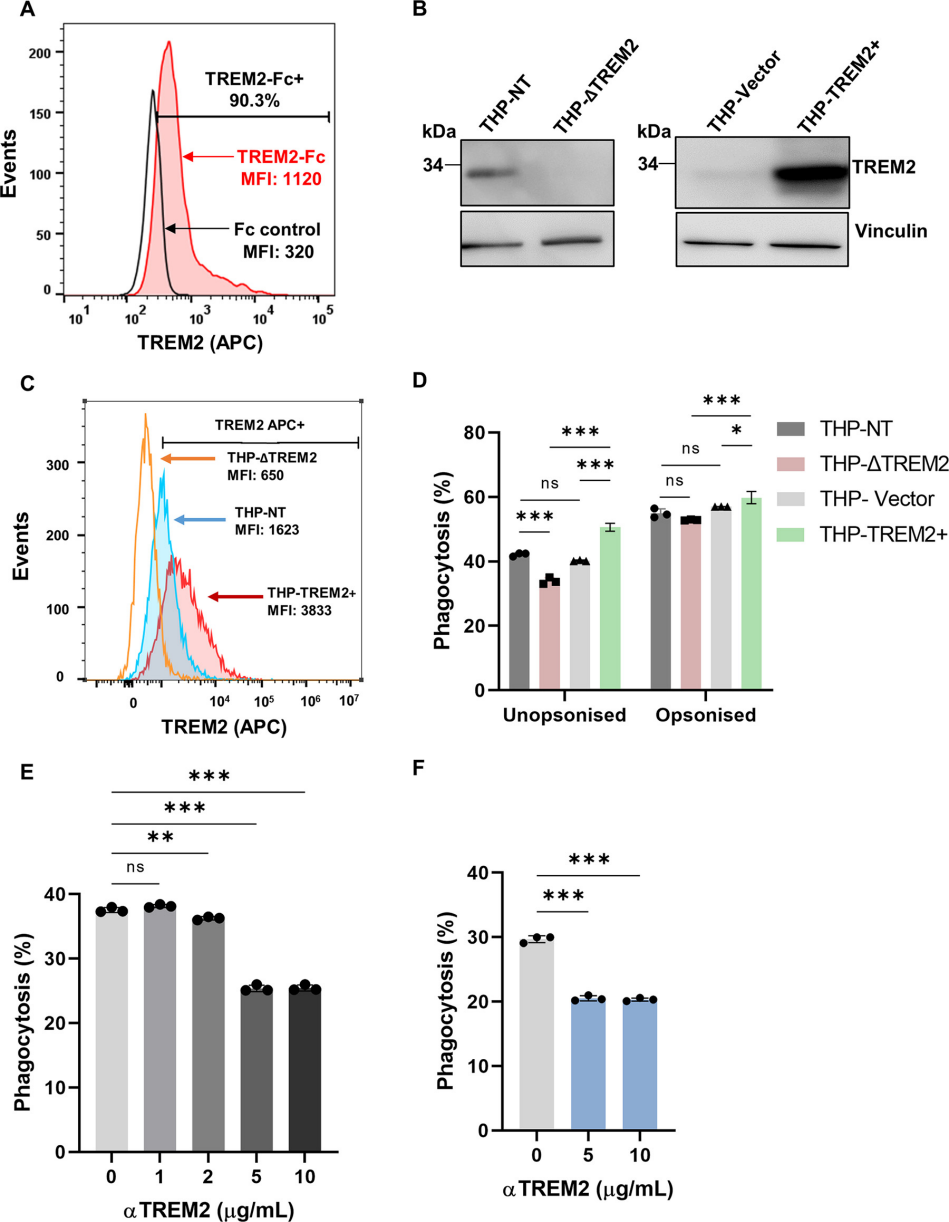
Human TREM2 binds to *M. tuberculosis* and contributes to phagocytosis. (A) *M. tuberculosis* was incubated with recombinant human TREM2-Fc or control Fc proteins. APC-conjugated anti-human IgG Fc antibody was used to detect the TREM2-bound bacteria. Stained bacteria were analyzed by flow cytometry, with APC^+^ signal indicating TREM2-Fc-bound bacteria. (B and C) THP-NT, THP-ΔTREM2, THP-Vector, or THP-TREM2+ cells were analyzed (B) for TREM2 protein expression by Western blotting or (C) for surface expression of TREM2 by staining with APC-conjugated anti-TREM2 antibody followed by flow cytometric analysis. (D) THP-1 and its derivative cell lines were incubated for 4 h with a GFP-expressing *M. tuberculosis* strain (Mtb-GFP) that had been either mock treated (unopsonized condition) or pretreated with human serum for 20 min (opsonized condition) to allow for phagocytosis. The amount of phagocytosis was quantified by flow cytometry, as indicated by the percentage of GFP^+^ macrophages. (E and F) Anti-TREM2 antibody was used at various concentrations (1 to 10 μg/mL) to treat (E) THP-1 cells or (F) hMDMs, followed by infection with Mtb-GFP. At 4 h postinfection, macrophages were analyzed by flow cytometry to quantify levels of phagocytosis. The results in this figure are representative of three biological replicates, and error bars indicate the mean ± SD from three independent experiments.

Mycobacteria are known to express a wide range of membrane lipids that are capable of binding and influencing the host immune responses of macrophages (32). A previous study revealed nonglycosylated mycobacterial cell wall mycolic acid as the ligand for murine TREM2 (31). Therefore, we sought to determine whether *M. tuberculosis* also binds to human TREM2. *M. tuberculosis* incubated with purified human TREM2-Fc in combination with anti-human IgG-allophycocyanin (APC) antibody showed a 90% shift in staining compared to control purified human Fc protein, which corresponded to an ~3-fold increase in the mean fluorescence intensity (MFI) of TREM2-Fc-stained *M. tuberculosis* (Fig. 1A), confirming that *M. tuberculosis* does bind to human TREM2. To study the function of this receptor in the phagocytosis and sensing of *M. tuberculosis*, we used THP-1 monocytes/macrophages. THP-1 is a human macrophage-like cell-line that is routinely used to study macrophage function due to its functional similarity to primary human monocyte-derived macrophages (hMDMs) (33). We generated THP-1 cell lines that either deleted TREM2 (THP-ΔTREM2) or overexpressed TREM2 (THP-TREM2+). In parallel, we generated THP-1 cells transduced with a vector expressing a non-target sgRNA sequence (THP-NT) or an empty vector (THP-Vector), which were used as control cells for the knockout and overexpressing cells, respectively, throughout this study. Western blot analysis and surface staining confirmed the deletion or overexpression of TREM2 in the genetically modified cell lines (Fig. 1B and C). To examine the role of TREM2 in *M. tuberculosis* infection, we used *M. tuberculosis* mc^2^6206, an auxotrophic strain derived from the virulent H37Rv strain that shares comparable intracellular responses with its parent (34). Deletion of TREM2 reduced the phagocytosis of *M. tuberculosis* by 13% of the total amount of phagocytosis measured in control THP-NT macrophages, whereas overexpression of TREM2 increased the phagocytosis of *M. tuberculosis* by 26% and 42% relative to THP-Vector and THP-ΔTREM2 macrophages, respectively (Fig. 1D). The effect of TREM2 on the uptake of *M. tuberculosis* was abrogated when the bacteria were opsonized, suggesting that TREM2 functions as a nonopsonic phagocytic receptor for *M. tuberculosis*. Using an antibody-mediated neutralization approach, we confirmed in both THP-1 macrophages and hMDMs that blocking of TREM2 with anti-TREM2 diminished the ability of the macrophages to phagocytose *M. tuberculosis* by 42% and 28% of the maximum phagocytosis in untreated THP-1 macrophages (Fig. 1E) or hMDMs (Fig. 1F), respectively. These results support the function of TREM2 in phagocytosis of *M. tuberculosis*, which contrasts with the recent study by Lizasa et al., where TREM2 deficiency did not affect the ability of murine bone marrow-derived macrophages (BMDMs) to phagocytose mycobacteria (31). This disparity could be due to differences in the expression of phagocytosis receptors between human and murine macrophages or due to differences between M. bovis BCG used in the previous study and *M. tuberculosis* in our current study. Phagocytosis is a complex process that is mediated through multiple receptors, and it is known that *M. tuberculosis* can exploit a variety of such receptors. As such, our results showing that deletion of one receptor (TREM2) does not completely abolish phagocytosis are consistent with expectations.

### *M. tuberculosis* upregulates expression of TREM2 in human macrophages.

**FIG 2:**
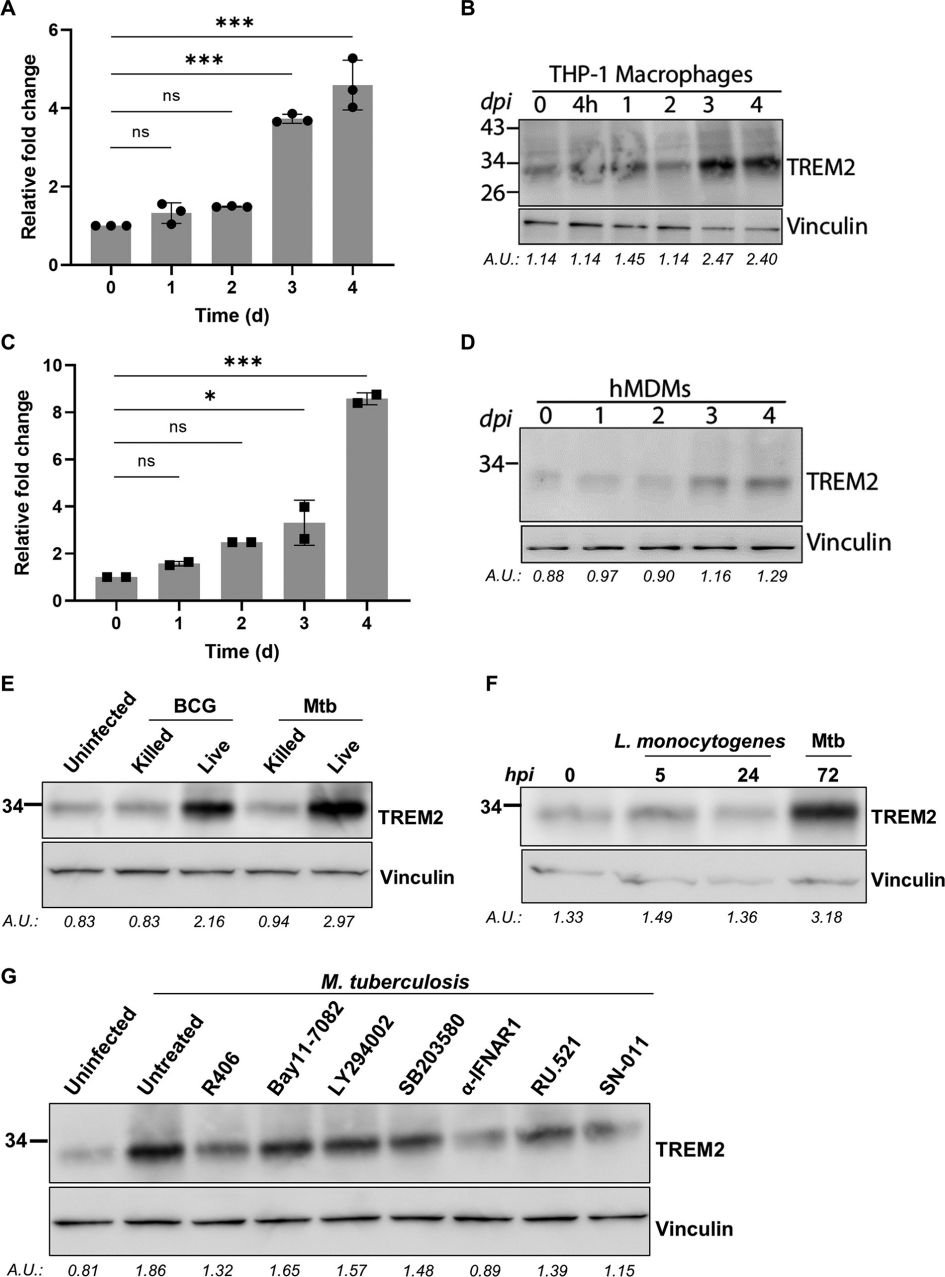
*M. tuberculosis* infection upregulates TREM2 expression. (A and B) *TREM2* mRNA and protein levels of *M. tuberculosis*-infected THP-1 macrophages (MOI = 10) were assessed by (A) qRT-PCR and (B) Western blotting at the indicated time points. hMDMs were infected with *M. tuberculosis* (MOI = 10) and at indicated time points postinfection, *TREM2* mRNA and protein levels were quantified by (C) qRT-PCR and (D) Western blot. Data shown in panels A and C were analyzed using the 2^−ΔΔ^*^CT^* method, normalized to *ACTB* as a reference gene, and are expressed as the relative fold change compared to uninfected cells. qPCR data represents the result of three technical replicates (mean ± SD). (E) M. bovis BCG and *M. tuberculosis* were mock treated or pretreated with gentamicin (150 μg/mL) for 1 h. Bacteria were then used to infect THP-1 macrophages, and TREM2 protein levels were analyzed at day 3. (F) Listeria monocytogenes-infected (MOI = 10) and *M. tuberculosis*-infected THP-1 macrophages at the indicated time points were analyzed by Western blotting, and vinculin was used as a loading control. (G) THP-1 macrophages were pretreated with the indicated inhibitors for 2 h and 6 h (in the case of SN-011) and subsequently infected with *M. tuberculosis* (MOI = 10). TREM2 expression was analyzed by Western blotting at 3 days postinfection using vinculin as a loading control. Blots in panels B, D, E, F, and G are representative of three independent biological replicates. The quantification of TREM2 expression is shown below each panel and is reported as normalized expression over vinculin. AU, arbitrary units.

Previous studies have shown that TREM2 expression is modulated by different pathogens or in different disease conditions to regulate innate immune responses elicited by the host (29, 35). Indeed, we show that there is a time-dependent increase in the expression of TREM2 at the mRNA and protein levels following *M. tuberculosis* infection of THP-1 macrophages (Fig. 2A and B) and hMDMs (Fig. 2C and D). The upregulation of TREM2 occurs 3 days postinfection and is only triggered by infection with live mycobacteria, as gentamicin-killed M. bovis BCG and *M. tuberculosis* failed to induce TREM2 expression (Fig. 2E). Complete cytosolic escape or *M. tuberculosis*-specific virulence factors do not appear to be required for triggering increased TREM2 expression, given that live *M. bovis* BCG infection induced TREM2 expression, despite not being able to escape the phagosome. Interestingly, infection with Listeria monocytogenes, a bacterium that translocates to the cytosol, only mildly increased TREM2 expression at 5 h postinfection, suggesting that mycobacterium-specific products and/or host factors activated at later phases of infection may be required to trigger the upregulation of TREM2 (Fig. 2F). To investigate the signaling pathway involved in triggering of TREM2 expression by *M. tuberculosis* infection, we examined the effect of multiple pharmacological inhibitors against different signaling pathways. Inhibitors against NF-κB (Bay11-7082), phosphatidylinositol 3-kinase (PI3K) (Ly294002), and p38 mitogen-activated protein kinase (MAPK) (SB203580) did not affect the increased expression of TREM2 induced by *M. tuberculosis* infection. In contrast, inhibition of proteins that contribute to type I IFN signaling, including STING (SN-011) and IFNAR1, blocked the upregulation of TREM2 at day 3 postinfection (Fig. 2G). Interestingly, inhibition of cyclic GMP-AMP synthase (cGAS) (RU.521) and SYK (R-406) only mildly blocked the upregulation of TREM2 (Fig. 2G), suggesting that other intracellular sensors may compensate for or participate in *M. tuberculosis*-induced upregulation of TREM2. Together, these data show that *M. tuberculosis* infection triggers a strong upregulation of TREM2 expression in macrophages through a mechanism dependent on type I IFN signaling and suggest that *M. tuberculosis* may exploit the increased expression of TREM2 for its survival postinfection.

### TREM2 alters macrophage cytokine production during *M. tuberculosis* infection.

**FIG 3:**
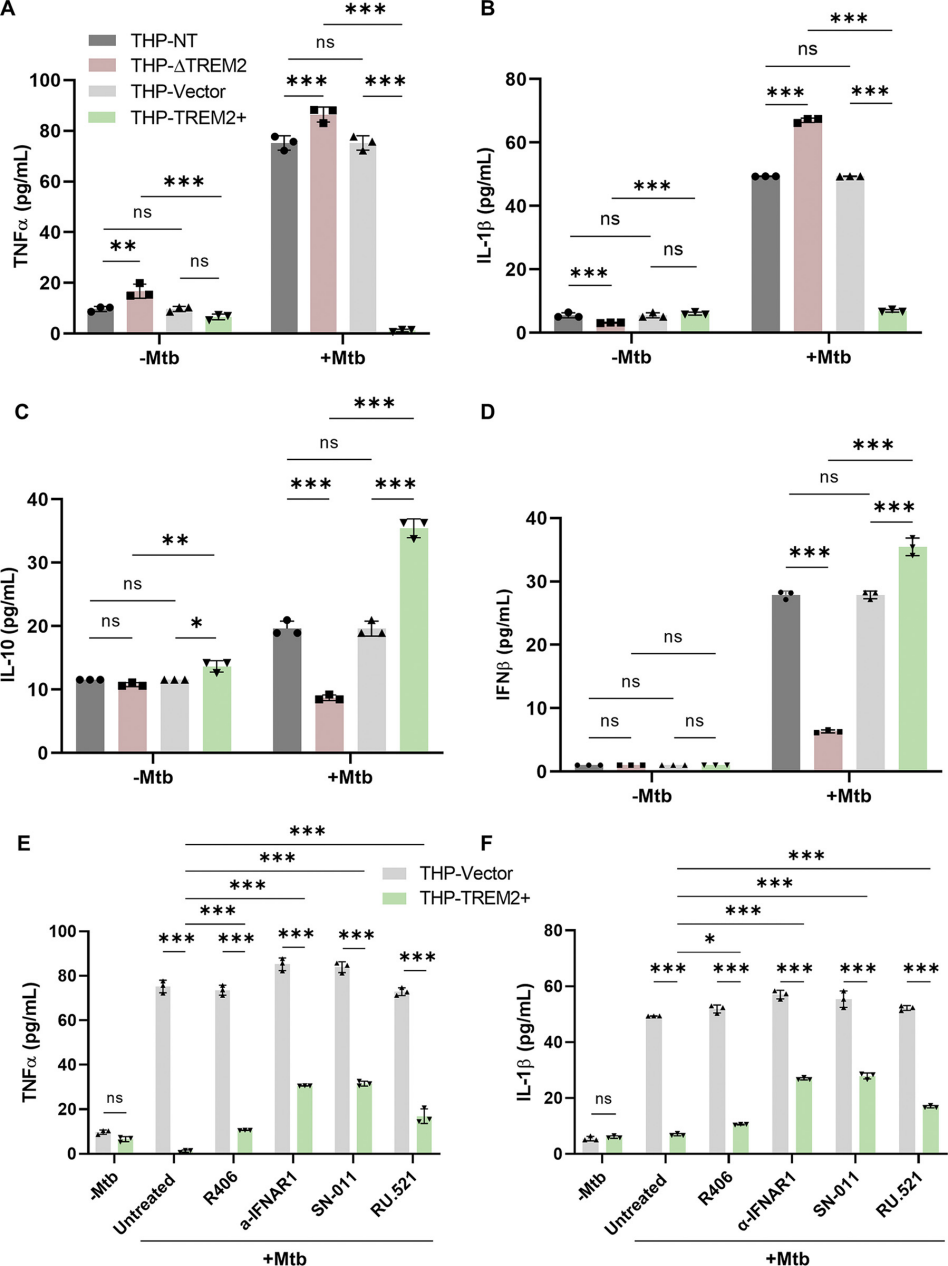
TREM2 expression alters cytokine production in *M. tuberculosis*-infected macrophages. (A to D) THP-NT, THP-ΔTREM2, THP-Vector, and THP-TREM2+ macrophages were infected with *M. tuberculosis* (MOI = 10), and culture supernatants were collected at 24 h postinfection. Levels of (A) TNF-α, (B) IL-1β, (C) IL-10, and (D) IFN-β were measured using human ELISA kits. (E and F) THP-Vector and THP-TREM2+ macrophages were mock treated or pretreated with R406 (SYK inhibitor; 10 μM), anti-IFNAR1 (2.5 μg/mL), SN-011 (STING inhibitor; 1 μM), and RU.521 (cGAS inhibitor; 10 μg/mL) for 2 h and 6 h (in the case of SN-011), prior to *M. tuberculosis* infection (MOI = 10). Culture supernatants were collected at 24 h postinfection and levels of (E) TNF-α and (F) IL-1β were measured. Error bars represent the mean ± SD of three independent biological replicates.

To determine whether the levels of TREM2 during *M. tuberculosis* infection affect macrophage function and inflammation, we examined cytokine production as a marker of the antibacterial response. THP-ΔTREM2, THP-TREM2+, and their respective control THP-NT and THP-Vector macrophages were infected with *M. tuberculosis*, and the levels of TNF-α, IL-1β, IL-10, and IFN-β in the culture supernatant were quantified at 24 h postinfection. Infected macrophages overexpressing TREM2 produced reduced levels of IL-1β and TNF-α, but increased levels of IL-10 and IFN-β, compared to the control cells (Fig. 3A to D). In contrast, infected macrophages lacking TREM2 produced increased levels of proinflammatory cytokines TNF-α and IL-1β, and reduced levels of IFN-β and anti-inflammatory cytokine IL-10 compared to the control cells (Fig. 3A to D). While TNF-α and IL-1β are important proinflammatory cytokines against mycobacterial infection, IL-10 and IFN-β are key inflammatory mediators that are responsible for disease progression (20, 36). To determine the signaling pathway responsible for regulating inflammation, THP-TREM2+ macrophages were treated with type I IFN inhibitors (SN-011, RU.521, and anti-IFNAR1) or SYK inhibitor R406. Consistent with previous reports (19), inhibition of type I IFN, but not SYK, partially restored the production of TNF-α and IL-1β in TREM2-overexpressing macrophages, indicating that TREM2-mediated regulation of cytokine production is driven, at least in part, by type I IFN signaling (Fig. 3E and F). In addition, THP-ΔTREM2 macrophages showed a significant reduction in the production of TNF-α and IL-1β during *M. tuberculosis* infection in the presence of inhibitors to PI3K, NF-κB, MEK, and p38 MAPK, suggesting that these signaling pathways are responsible for increased production of inflammatory cytokines when TREM2 is deleted (Fig. S1 A to B). Our results suggest that an increase in TREM2 levels is associated with decreased inflammation via type I IFN signaling, further supporting a function for TREM2 in downmodulating the inflammatory responses during *M. tuberculosis* infection.

10.1128/mbio.01456-22.1 FIG S1 Deletion of TREM2 promotes production of inflammatory cytokines via NF-κB, PI3K, and ERK1/2 pathways. (A and B) THP-NT and THP-ΔTREM2 macrophages were mock treated or pretreated with R406 (SYK inhibitor; 10 μM), Bay11-7082 (NF-κB inhibitor; 5 μM), Ly294002 (PI3K inhibitor; 10 μM), U0126 (MEK1/2 inhibitor; 10 μM), or SB203582 (p38 MAPK inhibitor; 5 μM) for 2 h. Cells were then infected with *M. tuberculosis* (MOI = 10), and culture supernatants were collected at 24 h postinfection. Levels of (A) TNF-α and (B) IL-1β, were measured using human ELISA assay kits. Error bars represent the mean ± SD from three independent biological replicates. Download FIG S1, PDF file, 0.4 MB. Copyright © 2022 Dabla et al. 2022 Dabla et al. https://creativecommons.org/licenses/by/4.0/ This content is distributed under the terms of the Creative Commons Attribution 4.0 International license.

### Deletion of TREM2 induces inflammatory cell death.

**FIG 4:**
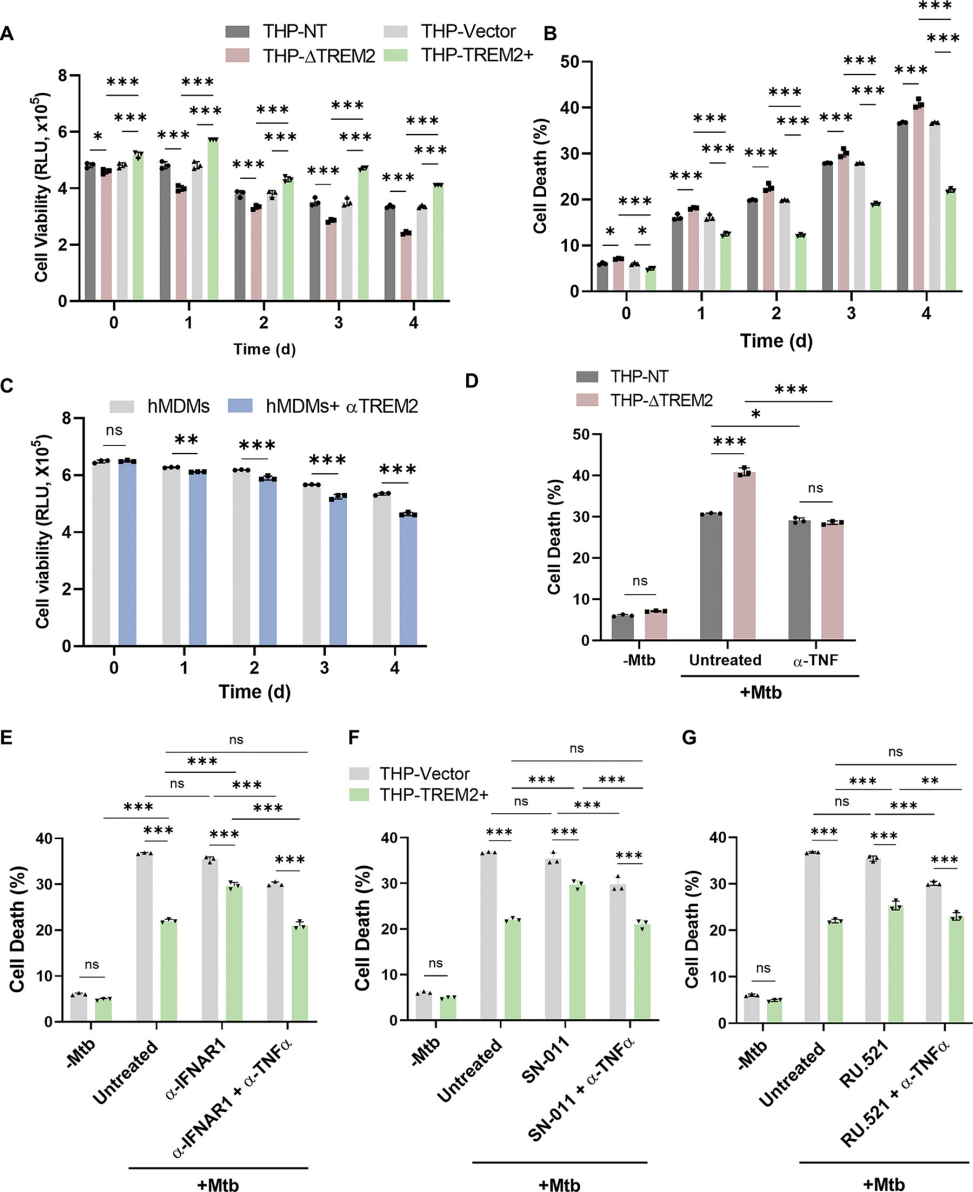
TREM2 regulates cell death through a mechanism dependent on type I IFN-mediated inhibition of TNF-α production. (A) THP-NT, THP-ΔTREM2, THP-Vector, and THP-TREM2+ macrophages were infected with *M. tuberculosis*, and cell viability was assessed using CellTiter-Glo (measured as relative luminescence units [RLU]). (B) THP-NT, THP-ΔTREM2, THP-Vector, and THP-TREM2+ macrophages were infected with *M. tuberculosis*, stained with FVS780 at the indicated time points, and analyzed using flow cytometry to measure the levels of cell death. (C) hMDMs mock treated or pretreated with anti-TREM2 antibody were infected with *M. tuberculosis* and assessed for cell viability using CellTiter-Glo. (D) THP-NT and THP-ΔTREM2 macrophages were mock treated or pretreated with 100 ng/mL anti-TNF-α antibody for 2 h. Cells were then infected with *M. tuberculosis*, and cell viability was quantified at day 4 postinfection by flow cytometry using FVS780 stain (12). THP-Vector, and THP-TREM2+ macrophages were mock treated or pretreated with (E) anti-IFNAR1 (2.5 μg/mL), (F) SN-011 (STING inhibitor; 1 μM), or (G) RU.521 (cGAS inhibitor; 10 μg/mL) for 2 h and 6 h (in the case of SN-011). Cells were then mock treated or pretreated with anti-TNF-α (100 ng/mL) for 2 h, followed by *M. tuberculosis* infection. The percentage of macrophage death was assessed at day 4 postinfection by staining with FVS780. An MOI of 10 was used for all infections in this figure. Error bars in this figure represent the mean ± SD from three independent biological replicates.

High levels of proinflammatory cytokines, such as TNF-α and IL-1β, are often associated with some form of cell death (37). Given that deletion of TREM2 resulted in increased production of inflammatory cytokines (Fig. 3), we hypothesized that TREM2 may regulate macrophage cell death during *M. tuberculosis* infection. Overall cell death was examined using the CellTiter-Glo assay, which measures cellular ATP using a luminescence-based luciferase reaction as a proxy for metabolically active and viable cells. *M. tuberculosis* infection of THP-ΔTREM2 macrophages resulted in increased cell death, as indicated by a decrease in luminescence compared to control macrophages (Fig. 4A). In contrast, overexpression of TREM2 protected macrophages from cell death during *M. tuberculosis* infection (Fig. 4A). To gain more clarity on the mode of cell death mediated by TREM2, we next used a LIVE/DEAD viability stain (FVS780), which provides information on the integrity of the plasma membrane. Consistent with results using the metabolic viability assay, deletion of TREM2 increased cell death by 5 to 10% relative to control macrophages, while overexpression of TREM2 enhanced cell viability by 10 to 15% (Fig. 4B). Importantly, neutralization of surface TREM2 on hMDMs with anti-TREM2 prior to *M. tuberculosis* infection reproduced the increase in cell death, as observed in THP-ΔTREM2 macrophages (Fig. 4C). These results suggest that reduced surface expression of TREM2 even prior to infection enables the bacteria to induce cell death and is independent of its expression levels postinfection. To delineate the mode of cell death, we utilized a panel of inhibitors to distinguish between apoptosis, pyroptosis, and necroptosis. THP-NT, THP-ΔTREM2, THP-Vector, and THP-TREM2+ macrophages were treated with z-VAD-FMK (zVAD), Nec-1s (38) or necrosulfonamide (39), and MCC950 (40), inhibitors that blocked apoptosis, necroptosis (39, 41), or the NLRP3 inflammasome, respectively, and subsequently infected with *M. tuberculosis*. Inhibitors of apoptosis or necroptosis did not block increased cell death in *M. tuberculosis*-infected ΔTREM2 macrophages (Fig. S2A), whereas inhibition of NLRP3 with MCC950 restored cell viability to the level of THP-NT control cells (Fig. S2B). We were able to confirm this finding in hMDMs with neutralized surface TREM2 upon treatment with MCC950 (Fig. S2C). Additionally, chemical induction of pyroptosis with lipopolysaccharide (LPS) and nigericin (42), triggered the same cell death profile in THP-1 macrophages with TREM2 deleted or overexpressed compared to their respective controls, THP-NT and THP-Vector macrophages, as well as in hMDMs (Fig. S2D and E). To investigate whether TREM2-mediated abrogation of TNF-α production is responsible for the enhanced viability in TREM2-overexpressing macrophages, we treated THP-ΔTREM2 macrophages with neutralizing antibody against TNF-α prior to *M. tuberculosis* infection. Neutralizing TNF-α antibody treatment indeed reduced cell death in THP-ΔTREM2 macrophages to the level of its control cells (Fig. 4D). Consistent with our cytokine results in Fig. 3E, treatment with either anti-IFNAR1, SN-011, or RU.521 to block type I IFN signaling enhanced the cell death in THP-TREM2+ macrophages, and this phenotype was reversed upon treatment with TNF-α neutralizing antibody (Fig. 4E to G). These results suggested that increased type I IFN signaling in TREM2-overexpressing macrophages is at least partially responsible for the enhanced viability in these macrophages during *M. tuberculosis* infection and that levels of TNF-α are the key contributor to cell death in our system.

10.1128/mbio.01456-22.2 FIG S2 Inhibition of NLRP3 restores cell viability in *M. tuberculosis*-infected THP-ΔTREM2 macrophages. (A and B) THP-NT, THP-ΔTREM2, THP-Vector, and THP-TREM2+ macrophages were mock treated or pretreated with (A) 40 μM z-VAD-FMK (zVAD), 10 μM Nec-1s, 10 μM necrosulfonamide (NSA), or (B) 0.3 μM MCC950 for 24 h. Cells were then infected with *M. tuberculosis* (MOI = 10), and cell viability was quantified using CellTiter-Glo. (C) hMDMs with or without anti-TREM2 treatment were mock treated or pretreated with 0.3 μM MCC950 and infected with *M. tuberculosis* (MOI = 10). Cell viability was analyzed using CellTiter-Glo. (D) THP-NT, THP-ΔTREM2, THP-Vector, and THP-TREM2+ macrophages or (E) hMDMs with or without anti-TREM2 treatment were mock treated or pretreated with LPS for 4 h prior to treatment with 5 μM nigericin for 24 h. The percentage of macrophage death was assessed by staining with FVS780. Error bars in this figure represent the mean ± SD from three independent biological replicates. Download FIG S2, PDF file, 0.3 MB. Copyright © 2022 Dabla et al. 2022 Dabla et al. https://creativecommons.org/licenses/by/4.0/ This content is distributed under the terms of the Creative Commons Attribution 4.0 International license.

### *M. tuberculosis* exploits TREM2 to promote its intracellular survival through a mechanism dependent on type I IFN signaling.

**FIG 5:**
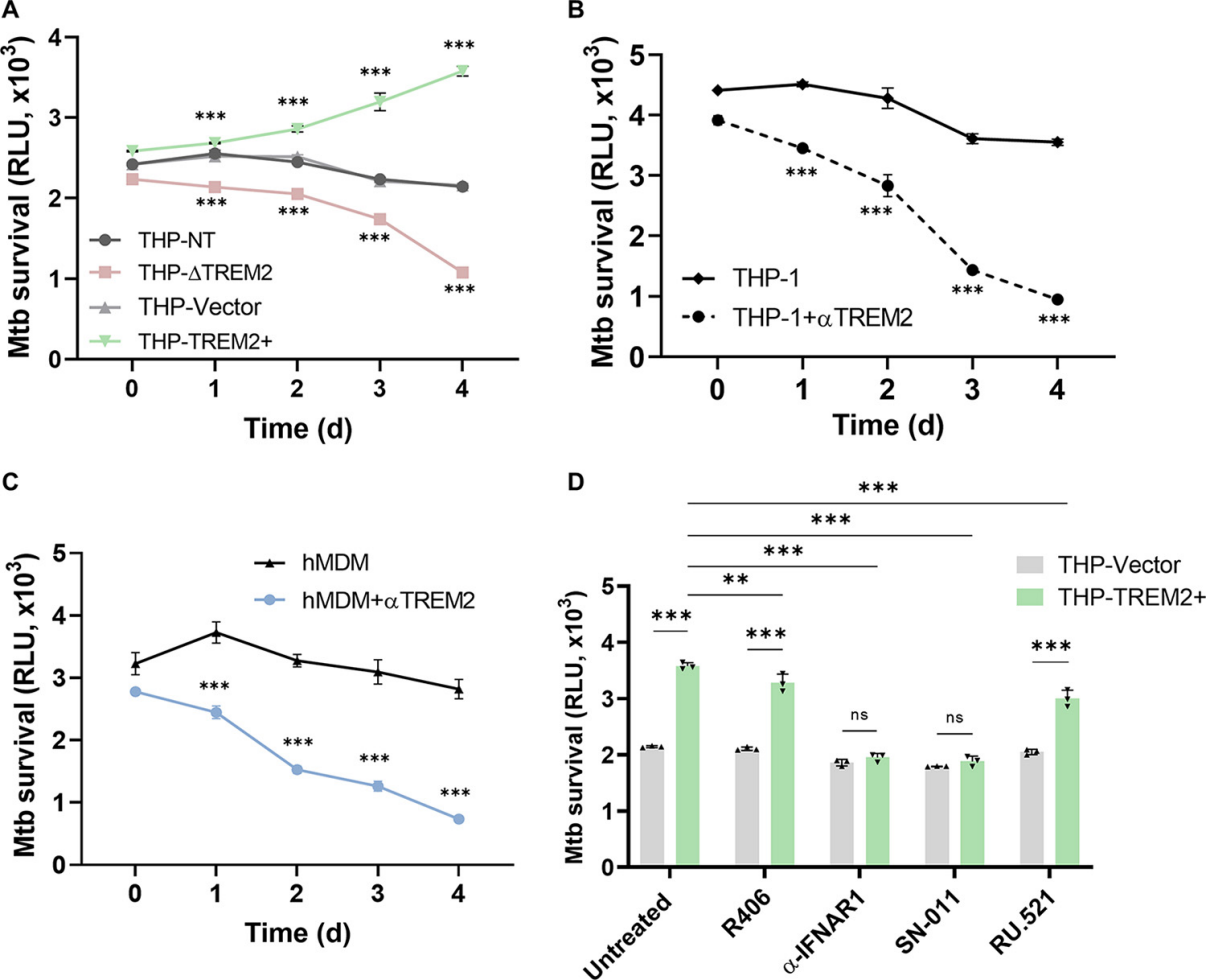
TREM2 facilitates intracellular survival of *M. tuberculosis*. (A) THP-NT, THP-ΔTREM2, THP-Vector, and THP-TREM2+ macrophages were infected with the autoluminescent Mtb-lux strain at an MOI of 10. Luminescence signal (RLU) representing viable *M. tuberculosis* was analyzed at the indicated time points. (B) THP-1 macrophages and (C) hMDMs were mock treated or pretreated with anti-TREM2 antibody for 20 min, followed by infection with Mtb-lux (MOI = 10), and RLU were analyzed at the indicated time points postinfection. (D) THP-Vector and THP-TREM2+ macrophages were mock treated or pretreated with R406 (SYK inhibitor; 10 μM), anti-IFNAR1 (2.5 μg/mL), SN-011 (STING inhibitor; 1 μM), or RU.521 (cGAS inhibitor; 10 μg/mL) for 2 h and 6 h (in the case of SN-011) and infected with Mtb-lux (MOI = 10). RLU were analyzed at day 4 postinfection. Error bars in this figure represent the mean ± SD from three independent biological replicates.

To address whether *M. tuberculosis*-induced upregulation of TREM2 expression impacts the outcome of infection, we investigated the intracellular *M. tuberculosis* burden in macrophages lacking or overexpressing TREM2. An autoluminescent *M. tuberculosis* reporter strain (Mtb-lux) expressing the *luxCDABE* operon was used to determine the intracellular bacterial burden during infection (43). Over a period of 4 days postinfection, THP-ΔTREM2 macrophages restricted the intracellular replication of *M. tuberculosis* and reduced bacterial survival compared to control THP-NT macrophages, whereas overexpression of TREM2 promoted increased intracellular *M. tuberculosis* replication compared to control THP-Vector macrophages (Fig. 5A). Differences in initial uptake of bacteria by the different THP-1 cell-lines could be visualized at day 0 (4 h postinfection) (Fig. 5A) and are consistent with the TREM2-mediated phagocytosis phenotype (Fig. 1). However, these differences only very slightly contribute to the much more drastic differences in bacterial survival by days 3 to 4 postinfection, indicating that TREM2-mediated effects on antibacterial activity are not simply due to differences in phagocytosis. Consistent with these results, neutralization of surface TREM2 in both THP-1 macrophages (Fig. 5B) and hMDMs (Fig. 5C) also inhibited the intracellular survival of *M. tuberculosis*. Collectively, these results indicate that *M. tuberculosis* may exploit TREM2 signaling to promote its survival inside the macrophage.

Master et al. showed that inflammasome activation results in enhanced phagosome maturation, increased *M. tuberculosis* clearance by macrophages, and reduced *M. tuberculosis* burden *in vivo* (44). To investigate whether inflammasome activation driven by the deletion of TREM2 is responsible for the decreased *M. tuberculosis* burden in THP-ΔTREM2 macrophages, we blocked the NLRP3 inflammasome with MCC950 in THP-ΔTREM2 macrophages and surface TREM2-neutralized hMDMs and evaluated bacterial survival. Inhibition of inflammasome activation did not restore intracellular *M. tuberculosis* survival in macrophages with TREM2 deleted or neutralized (Fig. S3A and B), indicating that the pyroptotic cell death induced by a lack of TREM2 signaling is not responsible for intracellular bacterial killing. These results suggest the existence of other TREM2-mediated antibacterial pathways or mechanisms that promote intracellular *M. tuberculosis* survival.

10.1128/mbio.01456-22.3 FIG S3 Enhanced bacterial clearance in TREM2-deficient or neutralized macrophages does not depend on NLRP3 activation. (A) THP-NT, THP-ΔTREM2, THP-Vector, and THP-TREM2+ macrophages, or (B) hMDMs with or without anti-TREM2 treatment were mock treated or pretreated with 0.3 μM MCC950 for 24 h and infected with Mtb-lux (MOI = 10). *M. tuberculosis* viability (RLU) were analyzed at day 4 postinfection. Error bars in this figure represent the mean ± SD from three independent biological replicates. Download FIG S3, PDF file, 0.3 MB. Copyright © 2022 Dabla et al. 2022 Dabla et al. https://creativecommons.org/licenses/by/4.0/ This content is distributed under the terms of the Creative Commons Attribution 4.0 International license.

Induction of type I IFN during *M. tuberculosis* infection has also been shown to promote bacterial infection and replication (20). Indeed, inhibition of type I IFN signaling at the level of STING and IFNAR1 reduced the intracellular bacterial survival in THP-TREM2+ macrophages to levels observed in THP-Vector control cells, while inhibition of SYK (R406) and cGAS (RU.521) only slightly reduced the intracellular *M. tuberculosis* burden compared to that in untreated control cells (Fig. 5D). These results demonstrate that type I IFN signaling plays the largest role in the compromised antibacterial response of macrophages overexpressing TREM2 and that SYK and cGAS are largely dispensable. These data suggest that intracellular sensors other than cGAS may be involved in the TREM2-STING signaling axis during *M. tuberculosis* infection.

### TREM2-mediated induction of type I IFNs disables ROS production.

**FIG 6:**
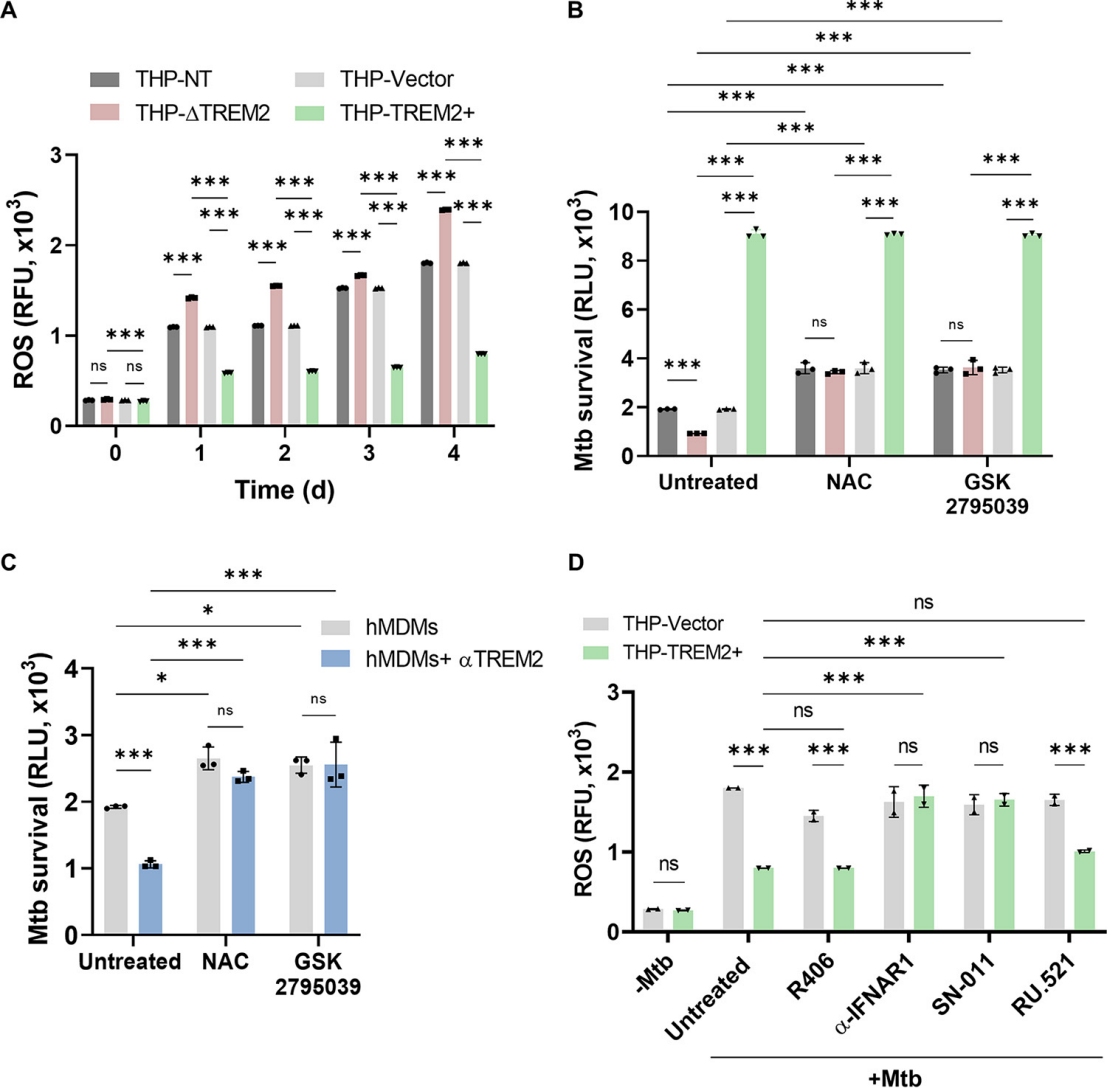
TREM2-mediated induction of type I IFNs disables ROS production (A) THP-NT, THP-ΔTREM2, THP-Vector, and THP-TREM2+ macrophages were infected with *M. tuberculosis* (MOI = 10) and stained with 5 μM DCFH-DA for 30 min at 37°C. ROS was quantified at the indicated time points postinfection by relative fluorescence unit (RFU) measurements. (B) THP-1 macrophages (THP-NT, THP-ΔTREM2, and THP-TREM2+) and (C) Mock or anti-TREM2 pretreated hMDMs were pretreated with 10 mM *N*-acetyl cysteine (NAC) or 25 μM GSK2795039 for 24 h, followed by infection with Mtb-lux (MOI = 10). RLU were analyzed at day 4 postinfection. (D) THP-Vector and THP-TREM2+ macrophages were mock treated or pretreated with R406 (SYK inhibitor; 10 μM), anti-IFNAR1 (2.5 μg/mL), SN-011 (STING inhibitor; 1 μM), or RU.521 (cGAS inhibitor; 10 μg/mL) for 2 h and 6 h (in the case of SN-011), prior to *M. tuberculosis* infection (MOI = 10). At day 4 postinfection, cells were stained with 5 μM DCFH-DA for 30 min at 37°C and ROS was quantified by fluorescence measurements. Error bars in this figure represent the mean ± SD from three independent biological replicates.

A major innate defense strategy that macrophages employ against *M. tuberculosis* is the production of ROS and RNI (37). Past studies have shown a conflicting role for TREM2 in regulating ROS production in different bacterial diseases (45, 46). TREM2 is involved in intracellular ROS production during infection with Salmonella enterica serovar Typhimurium or Pseudomonas aeruginosa (45, 47), whereas during Escherichia coli infection, overexpression of TREM2 resulted in decreased ROS (46). To delineate the role of TREM2 in regulating ROS production during *M. tuberculosis* infection, *M. tuberculosis*-infected macrophages were probed with the dye DCFH-DA (2′,7′-dichlorodihydrofluorescein diacetate) (48). THP-ΔTREM2 macrophages produced increased levels of ROS compared to control macrophages, while overexpression of TREM2 completely blocked the production of ROS (Fig. 6A). To evaluate whether the levels of ROS produced by macrophages with modulated TREM2 expression are directly linked to bacterial survival, we examined intracellular *M. tuberculosis* survival in the presence of inhibitors or scavengers of ROS. Treatment with *N*-acetyl cysteine (NAC) (49) or the specific NOX2 inhibitor GSK2795039 (50) restored the intracellular survival of *M. tuberculosis* in THP-ΔTREM2 macrophages to the levels in control macrophages (Fig. 6B). In addition, the reduction of *M. tuberculosis* survival in hMDMs with neutralized surface TREM2 was completely blocked by either NAC or GSK2795039, indicating that the ROS-mediated killing of intracellular *M. tuberculosis* was dependent on TREM2 signaling (Fig. 6C). Given our findings that type I IFNs play a role in *M. tuberculosis* survival in the context of TREM2 signaling (Fig. 5D), we speculated that type I IFNs may also be responsible for reduced ROS production mediated by increased expression of TREM2. Indeed, inhibition of IFNAR1 or STING resulted in enhanced production of ROS in THP-TREM2+ macrophages compared to untreated macrophages. In contrast, inhibition of cGAS or SYK did not alter ROS levels in THP-TREM2+ macrophages infected with *M. tuberculosis* (Fig. 6D), which was consistent with the minimal effects on *M. tuberculosis* survival in macrophages treated with the same inhibitors (Fig. 5D). These results demonstrate a mechanistic link between increased type I IFN signaling, decreased generation of ROS, and increased intracellular *M. tuberculosis* survival when TREM2 is upregulated. Interestingly, scavenging or blocking of intracellular ROS production by NAC or GSK2795039 also partially increased cell viability in THP-NT and THP-ΔTREM2 macrophages (Fig. S4A) as well as in untreated and anti-TREM2-treated hMDMs (Fig. S4B). Furthermore, chemical induction of pyroptosis by LPS and nigericin led to increased IL-1β production in THP-ΔTREM2 cells, which was partially blocked by treatment with NAC (Fig. S4C), suggesting that increased levels of cell death in macrophages with TREM2 deleted may be a consequence of increased ROS production. Taken together, our data show that TREM2 is a key receptor that is exploited by *M. tuberculosis* to suppress ROS production and macrophage cell death, thereby facilitating its survival within the host macrophage.

10.1128/mbio.01456-22.4 FIG S4 Inhibition of ROS partially restores cell viability in TREM2-deficient or -neutralized macrophages during *M. tuberculosis* infection. (A) THP-1 macrophages (THP-NT, THP-ΔTREM2, THP-Vector, and THP-TREM2+) and (B) hMDMs mock treated or pretreated with anti-TREM2 were mock treated or treated with *N*-acetyl cysteine (NAC) or GSK2795039 for 24 h, followed by infection with *M. tuberculosis* (MOI = 10). Macrophage viability was assessed at day 4 postinfection using CellTiter-Glo. (C) THP-NT, THP-ΔTREM2, THP-Vector, and THP-TREM2+ macrophages were mock treated or pretreated with NAC for 24 h. Cells were then primed with LPS for 4 h prior to treatment with 5 μM nigericin for 24 h. IL-1β was measured by ELISA. Error bars in this figure represent the mean ± SD from three independent biological replicates. Download FIG S4, PDF file, 0.3 MB. Copyright © 2022 Dabla et al. 2022 Dabla et al. https://creativecommons.org/licenses/by/4.0/ This content is distributed under the terms of the Creative Commons Attribution 4.0 International license.

## DISCUSSION

**FIG 7:**
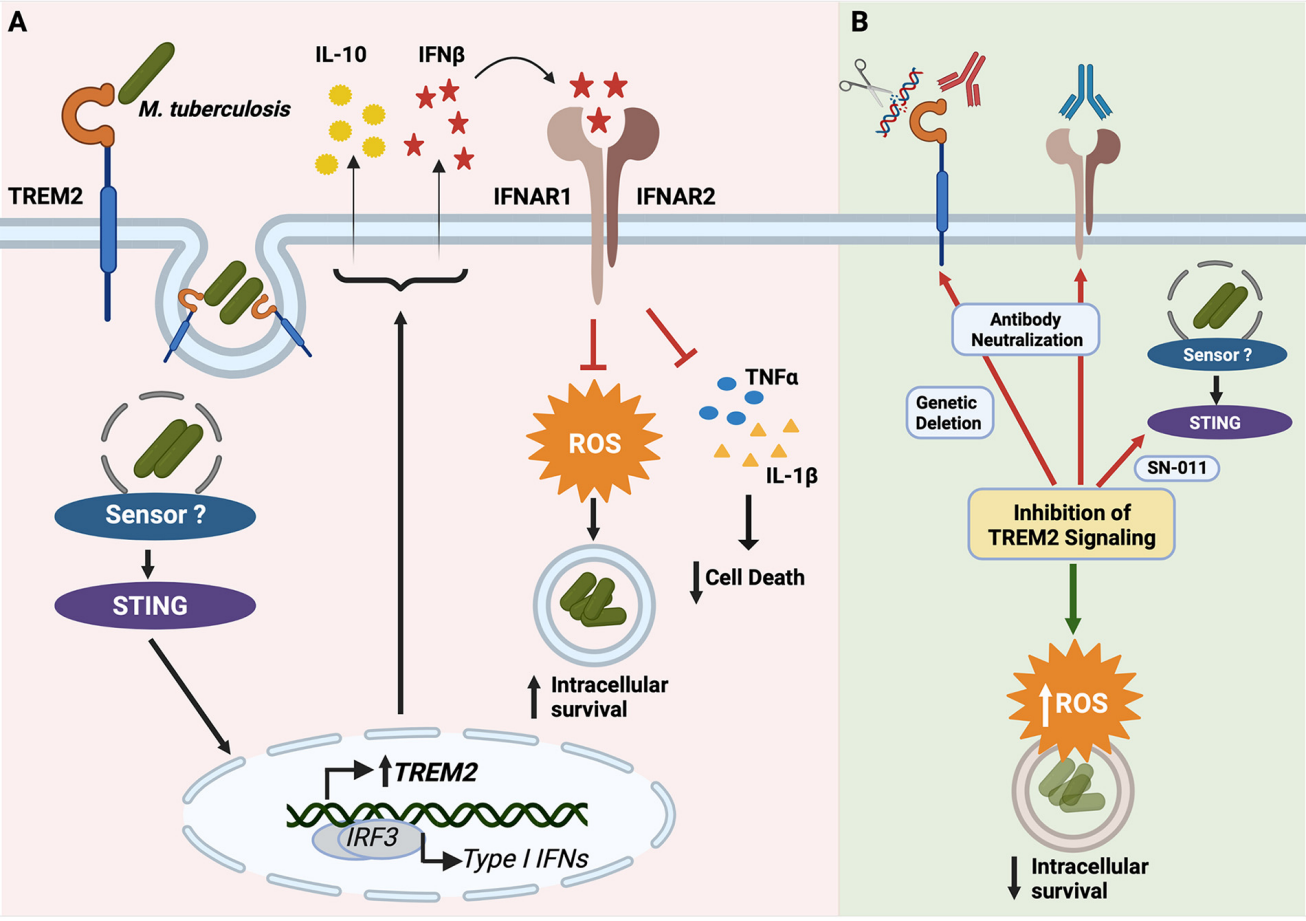
Role of TREM2 in macrophage antibacterial defense against *M. tuberculosis*. (A) Entry of *M. tuberculosis* via TREM2 induces a STING-dependent upregulation of TREM2 expression, which in turn increases IL-10 and IFN-β production. Increased levels of IFN-β is responsible for type I IFN-driven inhibition of ROS production and proinflammatory cytokine production, which results in the increased intracellular survival of *M. tuberculosis*. (B) Targeting the TREM2 signaling pathway by genetic deletion or antibody-mediated neutralization of TREM2, antibody-mediated neutralization of IFNAR1, or pharmacological inhibition of STING, restores proper proinflammatory cytokine and ROS production to promote bacterial clearance.

TREM2 is a receptor with important immunomodulatory functions that are mainly anti-inflammatory (22, 51, 52). Our study reveals a key role for TREM2 in the macrophage antibacterial response to *M. tuberculosis* infection (Fig. 7). TREM2 is predominantly expressed on the macrophage surface, where it recognizes and binds to different ligands, including anionic ligands present on bacterial surfaces (53), lipo-oligosaccharides (54), fibrillar amyloid β-associated lipids in the brain (55), and apolipoproteins (55). A recent study highlighted the role of TREM2 in mycobacterial infection and demonstrated mycolic acids such trehalose dimycolate, a mycobacterial glycolipid, to be ligands for murine TREM2 (31). However, it remained unclear whether TREM2 interacts with *M. tuberculosis* in human macrophages and if such interactions contribute to phagocytosis or an immunomodulatory role during infection. Our data demonstrate that human TREM2 does bind to *M. tuberculosis* and contributes to the phagocytosis of *M. tuberculosis* by macrophages (Fig. 1). That deletion of TREM2 does not completely abolish phagocytosis of *M. tuberculosis* was expected given that multiple receptors can contribute to the phagocytosis of *M. tuberculosis*, including mannose receptors (MRs) (56), complement receptor 3 (CR3) (56), Mincle (57), and scavenger receptors (MARCO) (58). While deletion of the *Trem2* gene in murine BMDMs did not affect phagocytosis of M. bovis BCG (31), this could be due to differences in the expression of alternative phagocytic receptors in murine macrophages. Our finding that TREM2 does contribute to phagocytosis of *M. tuberculosis* is consistent with reports that this receptor participates in the phagocytosis of apoptotic neurons (50), cellular debris (59) and other bacteria, such as E. coli and Streptococcus pneumoniae (30, 60).

We established that upregulation of TREM2 could only be induced by live mycobacteria and not gentamicin-killed bacteria (Fig. 2E), indicating that the response is dependent on an active process rather than just the surface accessible lipids as reported previously (31). Furthermore, we show that the STING and type I IFN pathways are essential for the upregulation of TREM2 (Fig. 2G), suggesting that sensing of mycobacterial DNA might be the trigger for regulation of TREM2 expression. However, the inhibition of cGAS failed to counteract the overexpression of TREM2 induced by *M. tuberculosis* infection, suggesting that other sensors might be involved in the process or might compensate for cGAS when it is pharmacologically inhibited. Alternatively, mycobacterially produced c-di-AMP may directly activate STING, a mechanism that would mirror L. monocytogenes activation of the STING pathway (18). Interestingly, L. monocytogenes infection only transiently and modestly induced TREM2 expression at early time points after infection (Fig. 2E), suggesting that additional mycobacterium-specific factors and/or unique host factors are likely required to trigger the upregulation of TREM2. Our result is consistent with the report that stimulation of the cGAMP-STING-IRF3 pathway induces the expression of TREM2 in Alzheimer's disease pathology (61). In addition, others have reported upregulation of TREM2 in response to porcine respiratory and reproductive syndrome virus (PRRSV) infection and polymicrobial sepsis (29, 35).

Since *M. tuberculosis* upregulates TREM2, we anticipated the possibility that TREM2 may be exploited by *M. tuberculosis* to promote bacterial survival. Engagement or avoidance of specific phagocytic receptors by *M. tuberculosis* offers a survival benefit for the bacteria. For example, phagocytosis of *M. tuberculosis* through MRs inhibits phagosome maturation (10), whereas engagement through CR3 or MARCO results in increased production of reactive oxygen (16) or inflammatory cytokines (62), respectively. TREM2 has been shown to function as a negative immunomodulator of inflammation and macrophage activation (52). Gao et al. reported an enhanced inflammatory response to LPS by alveolar macrophages when TREM2 was silenced (63). *In vivo* studies showed that TREM2^−/−^ mice showed increased activation of macrophages, as evidenced by elevated inflammatory cytokine production compared to wild-type mice (52). In our study, deletion of TREM2 intensified *M. tuberculosis*-induced proinflammatory cytokine production, whereas overexpression of TREM2 decreased proinflammatory cytokine and increased anti-inflammatory cytokine production (Fig. 3A to D). *M. tuberculosis* infection is known to induce production of type I IFNs that can lead to suppression of other cytokines, including TNF-α, which are critical for controlling infection (19). Recently, Korvatska et al. reported an unrecognized role of TREM2 as a positive regulator of the type I IFN response in myeloid cells (64). In line with this, we observed higher induction of IFN-β in TREM2-overexpressing macrophages, while TREM2-deficient macrophages showed reduced levels of IFN-β (Fig. 3D). In addition, pharmacological blockade of type I IFN signaling partially restored the production of proinflammatory cytokines in TREM2-overexpressing macrophages (Fig. 3E and F). In addition to altered cytokine profiles, we observed enhanced bacterial survival in TREM2-overexpressing macrophages, whereas deletion or antibody-mediated blocking of TREM2 increased bacterial clearance (Fig. 5A to C). Notably, this difference was not dependent on initial differences in phagocytosis as this only accounted for ~10 to 15% of the difference as observed at day 0. This suggests an important role for TREM2 signaling in promoting intracellular bacterial survival. Our data are in line with those from a previous report showing that knockdown of TREM2 accelerates *M. tuberculosis* clearance *in vivo* (31). Our results do not rule out the possibility that enhanced bacterial clearance in TREM2 knockout macrophages could be a result of increased immune responses via other receptor signaling, such as the Mincle signaling axis (31). Nevertheless, our data indicate that increased TREM2 signaling induces a type I IFN response responsible for reducing proinflammatory cytokine production and ROS generation, leading to increased intracellular survival of *M. tuberculosis*. Interestingly, the function of TREM2 in *M. tuberculosis* infection appears to be independent of SYK, the canonical downstream signaling kinase of TREM2, as inhibitors of SYK had little to no effect on ROS production or *M. tuberculosis* survival (Fig. 5D and Fig. 6D). However, SYK-independent signaling pathways of TREM2 have been reported (65). Our data also indicate that cGAS is largely dispensable for TREM2-mediated antimycobacterial functions (Fig. 5D and Fig. 6D), which is consistent with our finding that inhibition of cGAS does not affect upregulation of TREM2 induced by *M. tuberculosis* infection (Fig. 2G). Our finding that type I IFNs play a role in TREM2 signaling during *M. tuberculosis* infection is consistent with the known host-detrimental role of type I IFNs in animal models of *M. tuberculosis* infection (19, 20). However, type I IFN signaling is complex, and while it produces a host-detrimental response in many bacterial infections (e.g., Salmonella Typhimurium, L. monocytogenes, Staphylococcus aureus, M. tuberculosis, and Mycobacterium leprae), it has been proven to elicit enhanced antibacterial activity in macrophages in the context of S. pneumoniae, Helicobacter pylori, Streptococcus pyogenes, and polymicrobial sepsis (66). Similarly, there also remains conflicting evidence for the role of TREM2 in pathogen clearance. While deletion of TREM2 has been associated with increased pathogen clearance during infection with S. pneumoniae infection and PRRSV (29, 60), overexpression of TREM2 is responsible for reducing bacterial burden by exerting host antibacterial responses during polymicrobial sepsis and P. aeruginosa infection (35, 46). These contradictory findings regarding the role of TREM2 highlight the duality of its protective and detrimental functions in host defense, which may be linked to the type I IFN response produced by macrophages with elevated TREM2 expression.

The persistence of *M. tuberculosis* inside the macrophage is heavily influenced by the resulting mode of cell death. While apoptosis is associated with decreased *M. tuberculosis* growth (67), forms of cell death involving plasma membrane rupture, such as necrosis or sometimes pyroptosis, are often the result of uncontrolled intracellular replication of *M. tuberculosis* that subsequently promotes its dissemination through the extracellular milieu (68, 69). Our data show that TREM2 is responsible for controlling a small (10 to 15%), yet significant proportion of the overall observed macrophage cell death (Fig. 4A to C). Interestingly, we could not determine a specific mode of cell death regulated by TREM2 using an inhibitor-based approach, other than a mild effect observed by blocking the NLRP3 inflammasome. However, our data indicated that the cell death modulated by TREM2 depends on levels of TNF-α (Fig. 4D), which in turn are regulated by levels of type I IFN (Fig. 4E to G).

The generation of ROS by macrophages is a potent antibacterial response, but is actively disabled by *M. tuberculosis* infection via effector proteins such as Eis (enhanced intracellular survival) (70), Ndk (15), and NuoG (71), which counteract ROS production by inhibiting NOX2 or preventing its assembly in the phagosome. Recent work from our lab showed that *M. tuberculosis* downregulates the expression of the host focal adhesion kinase (FAK) to inhibit ROS production and enhance bacterial replication (72). The functional role of TREM2 in intracellular ROS production is contradictory. Studies have found TREM2 to be essential for intracellular bacterial killing by enhancing ROS production in the context of Salmonella Typhimurium (47) and P. aeruginosa (45) infection. In contrast, evidence also exists that TREM2 suppresses ROS levels in murine models during E. coli infection (46). In the context of *M. tuberculosis* infection, we found that elevated TREM2 expression blocks ROS production to promote intracellular *M. tuberculosis* survival (Fig. 6A). Consistent with this, increased ROS production by TREM2-deficient macrophages is responsible for the enhanced *M. tuberculosis* clearance as scavenging of ROS or inhibition of NOX2 restores the *M. tuberculosis* burden in these cells (Fig. 6B and C). However, scavenging of ROS in TREM2-overexpressing macrophages did not increase the *M. tuberculosis* burden beyond that of untreated TREM2+ macrophages (Fig. 6B). This could be explained by the low levels of ROS produced by TREM2-overexpressing macrophages, wherein the maximum levels of intracellular *M. tuberculosis* burden have already been attained and any further reduction in ROS production does not lead to a detectable difference in intracellular *M. tuberculosis* replication. Interestingly, a recent study showed that type I IFNs shift macrophage metabolism to aerobic glycolysis in inflammatory macrophages and blocked ROS production during *M. tuberculosis* infection (20). Our study corroborates this finding as inhibition of type I IFN signaling enhanced ROS production in TREM2-overexpressing macrophages comparable to that in control cells (Fig. 6D).

Collectively, our study demonstrates the importance of TREM2 both as a phagocytic receptor and as a signaling hub for the macrophage antibacterial response to *M. tuberculosis* infection (Fig. 7). While there are no current therapies in preclinical or clinical trials targeting TREM2 in the context of TB, AL002, a humanized monoclonal TREM2 antibody currently in a phase 2 clinical trial, has been reported to be a promising candidate for Alzheimer’s disease therapy (73). Given that data from our study and others (31) support the translational potential of targeting TREM2, the use of TREM2 neutralizing antibodies could be a promising option to boost our cellular antibacterial response to *M. tuberculosis* infection. As such, targeting TREM2 signaling could prove to be a potential therapeutic strategy against tuberculosis.

## MATERIALS AND METHODS

### Cell culture and reagents.

THP-1 monocytes (ATCC TIB-202), their derivative cell lines, and the peripheral blood mononuclear cells (PBMCs) used in this study were cultured and maintained in RPMI 1640 medium (Gibco, Gaithersburg, MD). HEK 293T (ATCC CRL-11268) and HEK GP2-293 (Clontech-TaKaRa) cells were maintained in Dulbecco’s modified Eagle’s medium (DMEM) (Gibco). The medium was supplemented with 2 mM l-glutamine, penicillin-streptomycin (100 IU/mL penicillin and 100 μg/mL streptomycin), 10 mM HEPES, and 10% heat-inactivated fetal bovine serum (FBS). Cells were maintained at 37°C in a humidified atmosphere of 5% CO_2_. To obtain THP-1 macrophages, THP-1 monocytes were differentiated using 100 ng/mL phorbol 12-myristate 13-acetate (PMA) (Alfa Aesar) for 72 h. Human peripheral mononuclear cells were collected as per the approved ethics protocol (2005388-01H) and isolated from the buffy coat using the Ficoll-Paque density centrifugation method. Monocytes were positively selected using anti-CD14-coated magnetic particles from StemCell Technologies (Vancouver, BC, Canada) as per the manufacturer’s protocol. To obtain human monocyte-derived macrophages (hMDMs), monocytes were differentiated using 5 ng/mL granulocyte-macrophage colony-stimulating factor (GM-CSF) (Gibco) for 6 days. The cell death inhibitors necrostatin-1s (Nec-1s) and z-VAD-FMK were purchased from New England Biolabs (Ipswich, MA) and Selleckchem, respectively. Necrosulfonamide (74), *N*-acetyl cysteine (NAC), and GSK2795039 were purchased from Millipore Sigma. Puromycin was purchased from Gibco. Inhibitors against different signaling pathways R406, Bay11-7082, Ly294002, U0126, SB203580, RU.521, and SN-011 were purchased from Cedarlane.

### Bacterial strains and plasmids.

The derivative auxotroph strain mc^2^6206 (Δ*panCD* Δ*leuCD*) of *M. tuberculosis* H37Rv was grown in Middlebrook 7H9 medium (BD Biosciences) supplemented with 0.2% glycerol (Fisher Chemical), 0.05% Tween 80 (Acros Organics), 10% OADC (oleic acid-albumin-dextrose-catalase) (BD Biosciences), 24 μg/mL pantothenate (Thermo Fisher Scientific, Waltham, MA), and 50 μg/mL l-leucine (Alfa Aesar) or plated on Middlebrook 7H10 plates supplemented with 0.5% glycerol and 10% OADC. The green fluorescent protein (GFP)-expressing mc^2^6206 *M. tuberculosis* strain (Mtb-GFP) was generated previously (75). The autobioluminescent Mtb-lux strain was generated by transforming mc^2^6206 with the pMV306hsp+LuxG13 plasmid. pMV306hsp+LuxG13 plasmid was a gift from Brian Robertson and Siouxsie Wiles (Addgene plasmid 26161 [http://n2t.net/addgene:26161]; RRID, Addgene_26161) (43). The Mtb-GFP and Mtb-lux strains were maintained in a 7H9 selection medium containing 50 μ/mL hygromycin B (Calbiochem) or 25 μ/mL kanamycin, respectively. All liquid *M. tuberculosis* cultures were maintained at 37°C with slow shaking (50 rpm). E. coli strains DH5α and NEB stable (New England Biolabs) were used to propagate the plasmids. The pMSCV-TREM2 plasmid was constructed by PCR amplifying the *TREM2* gene from THP-1-derived cDNA using the oligonucleotide pairs flanked by the XhoI and HpaI restriction sites, respectively, before cloning into the multiple-cloning sites of pMSCV-puro (Clontech) for retrovirus-mediated expression. The CRISPR-Cas9 knockout plasmid targeting human *TREM2* (pSL106) and non-targeting control plasmid (pSL20) were generated using the annealed single guide RNA (sgRNA) oligonucleotide 5′-ACTGGTAGAGACCCGCATCA-3′ (64) and 5′-GTACCATACCGCGTACCCTT-3′, respectively. The sgRNAs were inserted into the modified LentiCRISPRv2 vector (pSL50) at the BsmBI restriction sites, as previously described (76). All plasmids were validated by Sanger DNA sequencing.

### Generation of THP-1 derivative cell lines.

Lentiviral supernatant was produced by transfecting HEK 293T cells with the generated plasmids (pSL106 or pSL20 [78]), pVSV-G envelope plasmid, and the psPAX2 packaging plasmid (a gift from Didier Trono, Addgene plasmid 12260 [http://n2t.net/addgene:12260]; RRID, Addgene_12260) using FuGENE (Promega). Retroviral plasmids (pMSCV-puro and pMSCV-TREM2) were cotransfected with the envelope plasmid pVSVG into HEK GP2-293 cells using FuGENE. Supernatants containing viral particles were either used immediately for transduction or stored at −80°C. For transduction, viral supernatants were supplemented with 10 μg/mL DEAE-dextran (Sigma-Aldrich) and added to THP-1 cells. After 72 h, the transduced cells were selected using puromycin (1 μg/mL) and analyzed by flow cytometry and Western blotting to verify deletion or overexpression of TREM2.

### TREM2-*M. tuberculosis* binding assay.

Fifty million Mtb-GFP cells were fixed in phosphate-buffered saline (PBS) with 0.05% Tween 80 and 2% paraformaldehyde (Thermo Fisher Scientific) for 30 min. For staining, either 2 μg TREM2-Fc or control Fc protein (gift from Aaron Ring) was incubated with the Mtb-GFP for 30 min at room temperature. Bacterial cells were then incubated with 2 μg of Alexa Fluor 647 anti-human IgG Fc antibody (BioLegend) for another 15 min. Cells were washed and then analyzed using flow cytometry. *M. tuberculosis* cells were gated based on scatter and GFP expression, while the APC channel was used for detection of TREM2-Fc or Fc binding signals.

### Macrophage infections.

THP-1 macrophages or hMDMs were incubated in RPMI infection medium without antibiotic for 1 to 2 h before infection. *M. tuberculosis*, Mtb-GFP, or Mtb-lux (as per the experiment) were grown to log phase and added to the cells at a multiplicity of infection (MOI) of 10 (10 bacteria to 1 cell), using the conversion of 3 × 10^8^ bacteria/mL at an optical density at 600 nm (OD_600_) of 1.0. After 4 h, nonphagocytosed extracellular bacteria were removed by PBS washes, and plates were placed back at 37°C for the desired time frame. When infection was performed in the presence of z-VAD-FMK, necrosulfhonamide (74), Nec-1s, MCC950, *N*-acetyl cysteine, or GSK2795039, the cells were pretreated for 24 h before infection. For infection in the presence of R406, Bay11-7082, Ly294002, U0126, SB203580, or RU.521, cells were pretreated for 2 h and for SN-011, cells were pretreated for 6 h, prior to infection. To block TREM2 signaling, cells were pretreated with a human/mouse APC-conjugated anti-TREM2 antibody (FAB17291A; R&D Systems) for 20 min at room temperature before infection. To block type I signaling and TNF-α, cells were pretreated with anti-IFNAR1 (MAB1155; Millipore Sigma) and human TNF-α neutralizing antibody (7321S; New England Biolabs) for 30 min and 2 h, respectively, prior to infection.

### Phagocytosis and intracellular *M. tuberculosis* survival assay.

hMDMs or THP-1 macrophages seeded at a density of 75,000 cells per well were infected with Mtb-GFP as described above. For opsonized samples, the bacteria were pretreated with 20% human serum for 30 min before infection. After 4 h, extracellular bacteria were removed by PBS washes. Cells were detached from the wells using TrypLE Express (Gibco), resuspended in PBS with 0.01% FBS, and GFP signal corresponding to intracellular bacteria was analyzed by flow cytometry. To assess the intracellular survival of *M. tuberculosis*, macrophages were infected with Mtb-lux as described above, and luminescence signal representing viable intracellular bacteria was measured at the desired time points using the Synergy H1 microplate reader (BioTek), with an optimized integration time of 10 s.

### Macrophage viability assay.

**(i) FVS780 LIVE/DEAD assay.** The BD Horizon fixable viability stain 780 (FVS780) assay (BD Biosciences) was used to determine plasma membrane permeability. Culture supernatants were preserved for analysis of detached cells. Cells were then washed with PBS and detached with TrypLE Express. Harvested cells were washed with PBS and stained with BD Horizon FVS 780 as per the manufacturer’s protocol. Viable and dead cells were then analyzed and quantified using flow cytometry.

**(ii) CellTiter-Glo assay.** The CellTiter-Glo system (Promega) was used as per the manufacturer’s protocol to determine the number of viable cells based on their metabolic activity. At the indicated time points postinfection, CellTiter-Glo reagent was added to each well in equal volumes. Contents were mixed for 2 min to induce cell lysis, followed by a 10-min incubation to stabilize the luminescence signal. The resultant luminescence was measured using the Synergy H1 microplate reader in 96-well solid white plates with an integration time of 1 s per well.

### Flow cytometry.

Flow cytometric analysis was performed using the CytoFLEX flow cytometer (Beckman Coulter, Indianapolis, IN), and data were analyzed using the CytExpert software (Beckman Coulter) and FlowJo V10 software (BD Biosciences). For surface expression analysis, THP-1 macrophages or hMDMs were stained at 4°C for 30 min with human/mouse APC-conjugated anti-TREM2 (FAB17291A; R&D Systems). Flow cytometry was also performed to measure the density and viability of cultured cells and phagocytosis levels in macrophages.

### Cytokine profiling.

Cell culture supernatants were harvested from control, noninfected, and *M. tuberculosis*-infected THP-1 or THP-1-derived macrophages, and cytokine expression was measured at 24 h postinfection. Human enzyme-linked immunosorbent assay (ELISA) kits were used as per the manufacturer’s protocol to measure the expression of TNF-α, IL-1β, IL-10 (Invitrogen, Carlsbad, CA), and IFN-β (R&D Systems). The absorbance of the samples was read at 450 nm using the Synergy H1 microplate reader.

### Quantitative real-time PCR.

Total RNA was isolated from THP-1 or hMDMs using the Aurum total RNA minikit from Bio-Rad (Hercules, CA). Five hundred nanograms of total RNA was used to synthesize cDNA using the iScript reverse transcription supermix (Bio-Rad). Gene expression of *TREM2* along with that of the reference gene *ACTB* was analyzed by quantitative real-time PCR (qRT-PCR) with the CFX96 Touch real-time PCR detection system (Bio-Rad) using custom primers designed based on MIQE guidelines (77). The threshold cycle (2^−ΔΔ^*^CT^*) (79) method was used to determine relative gene expression normalized to *ACTB*. Relative fold changes in expression were normalized to uninfected macrophages (day 0) set as 1.0. The following primer pairs were used: human *TREM2*, 5′-GGCTGCTCATCTTACTCTTTG-3′ and 5′-GAGTCATAGGGGCAAGACAC-3′; human *ACTB*, 5′-ATTGCCGACAGGATGCAGAA-3′ and 5′-GCTGATCCACATC TGCTGGAA-3′.

### Immunoblotting.

Macrophages were harvested, washed with PBS, and lysed in radioimmunoprecipitation assay (RIPA) buffer (150 mM NaCl, 1% IGEPAL CA-630, 0.5% sodium deoxycholate, 0.1% SDS, 50 mM Tris [pH 8.0]) containing protease and phosphatase inhibitors (Halt protease and phosphatase inhibitor cocktail; Thermo Fisher Scientific) as per the manufacturer’s protocol. The protein concentration of the lysates was determined using the RC DC protein assay kit (Bio-Rad) according to the manufacturer’s protocol. About 35 μg of protein per sample was separated by SDS-PAGE using handcast gels (SureCast Gel Handcast System, Invitrogen) and transferred onto a polyvinylidene difluoride (PVDF) membrane using a wet transfer system (Mini-Protean Tetra vertical electrophoresis cell; Bio-Rad). Primary monoclonal rabbit TREM2 antibody (D8I4C, Cat. #91068) was purchased from Cell Signaling Technologies. Primary monoclonal mouse glyceraldehyde-3-phosphate dehydrogenase (GAPDH) antibody (GA1R) was purchased from Invitrogen. Horseradish peroxidase (HRP)-conjugated goat anti-rabbit or goat anti-mouse polyclonal antibodies (Bio-Rad) were used as secondary antibodies. Blots were developed using the Clarity Western enhanced chemiluminescence substrate (Bio-Rad). Imaging analysis was performed with the ImageQuant LAS4000 imaging system (GE Healthcare Lifesciences, Cytiya).

### Reactive oxygen species assay.

The intracellular ROS level was measured by staining the cells with DCFH-DA (2′,7′-dichlorofluorescein diacetate) (Sigma-Aldrich) for 30 min at 37°C at the indicated time points postinfection. Stained cells were washed with PBS, and fluorescence intensity was measured at excitation/emission wavelengths of 485/535 nm using the Synergy H1 microplate reader.

### Statistical analysis.

All data are expressed as the mean ± standard deviation (SD) of results from three independent biological replicates. The significance of the results was analyzed using two-way analysis of variance (ANOVA), except for Fig. 2A and C, where one way ANOVA was performed. *P* values of <0.05 were considered significant (*, *P* < 0.05; **, *P* < 0.01; ***, *P* < 0.001). Results were plotted using GraphPad Prism version 9 (Graph Pad Prism Software, San Diego, CA).

### Data availability.

The source data for all the figures can be obtained from the corresponding author upon request.
