# Supplementary material for: TREM2 Promotes Immune Evasion by Mycobacterium tuberculosis in Human Macrophages
Source: mBio. 2022 Aug 4;13(4):e01456-22. doi: 10.1128/mbio.01456-22 (PMC9426521; doi:10.1128/mbio.01456-22)
Supplement: FIG S1 [file mbio.01456-22-sf001.pdf]

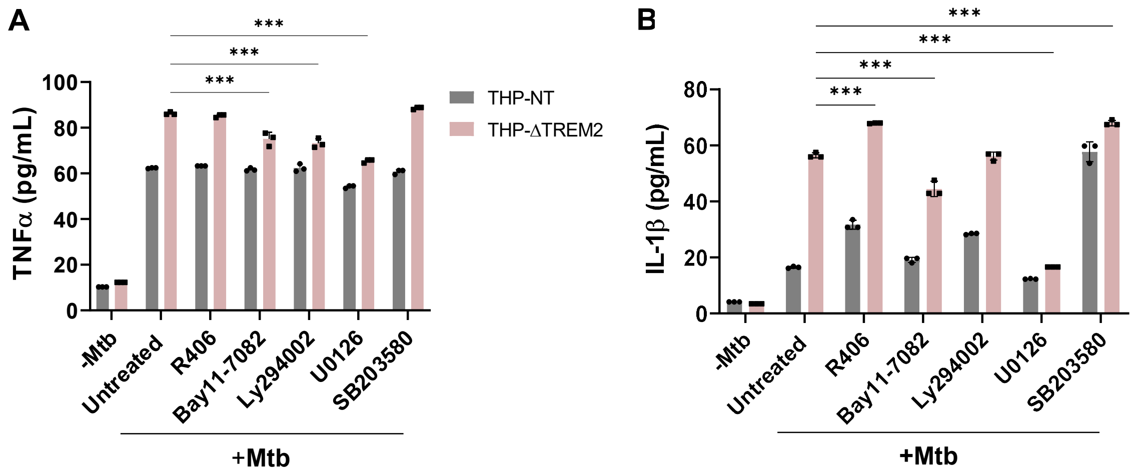

Figure S1. Deletion of TREM2 promotes production of inflammatory cytokines via NF-kB, PI3K and ERK1/2 pathways. (A, B) THP-NT and THP-ΔTREM2 macrophages were mock or pre-treated with R406 (Syk inhibitor, 10 μM), Bay11-7082 (NF-κB inhibitor, 5 μM), Ly294002 (PI3K inhibitor, 10 μM), U0126 (MEK1/2 inhibitor, 10 μM), and SB203582 (p38 MAPK inhibitor, 5 μM) for 2h. Cells were then infected with Mtb (MOI 10), and culture supernatants were collected at 24 h post-infection. Levels of **(A)** TNFα, and **(B)** IL-1β, were measured using a human ELISA assay kit. Error bars represent the mean ± SD of three independent biological replicates.
