# Supplementary material for: TREM2 Promotes Immune Evasion by Mycobacterium tuberculosis in Human Macrophages
Source: mBio. 2022 Aug 4;13(4):e01456-22. doi: 10.1128/mbio.01456-22 (PMC9426521; doi:10.1128/mbio.01456-22)
Supplement: FIG S2 [file mbio.01456-22-sf002.pdf]

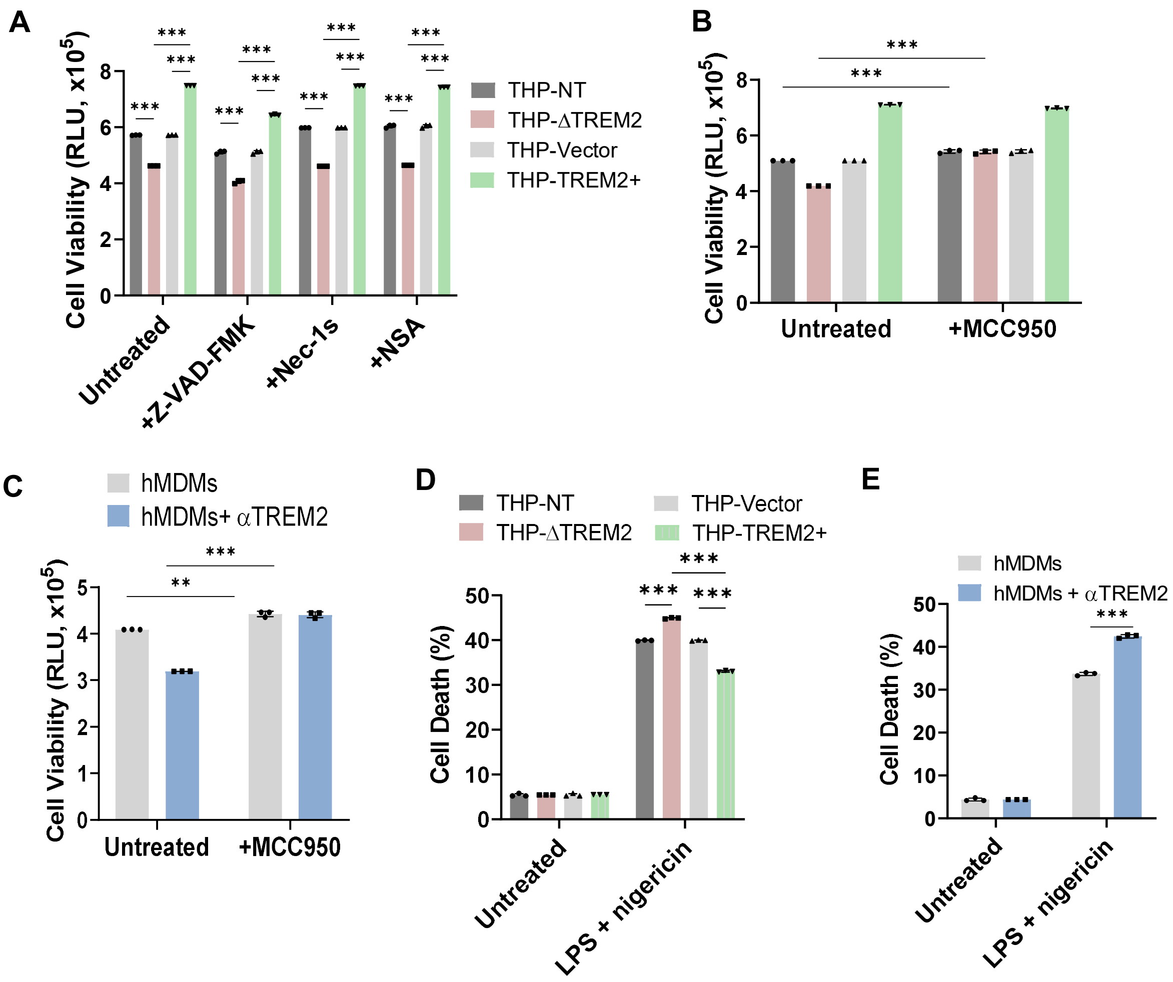

Figure S2. Inhibition of NLRP3 restores cell viability in Mtb-infected THP-ΔTREM2 macrophages. (A, B) THP-NT, THP-ΔTREM2 and THP-TREM2+ macrophages were mock-treated or pre-treated with **(A)** 40 μM z-VAD-FMK (zVAD), 10 μM Nec-1s, 10 μM necrosulphonamide (NSA), or **(B)** 0.3 μM MCC950 for 24 h. Cells were then infected with Mtb and cell viability was quantified using CellTiter-Glo. **(C)** hMDMs with or without anti-TREM2 treatment were mock-treated or pre-treated with 0.3 μM MCC950 and infected with Mtb (MOI 10). Cell viability was analysed using CellTiter-Glo. **(D)** THP-NT, THP-ΔTREM2, and THP-TREM2+ macrophages, or **(E)** hMDMs with or without anti-TREM2 treatment, were mock-treated or pre-treated with LPS for 4 h prior to treatment with 5 μM nigericin for 24 h. Percentage of macrophage death was assessed by staining with FVS780. Error bars in this figure represent the mean ± SD of three independent biological replicates.
