# Supplementary material for: TREM2 Promotes Immune Evasion by Mycobacterium tuberculosis in Human Macrophages
Source: mBio. 2022 Aug 4;13(4):e01456-22. doi: 10.1128/mbio.01456-22 (PMC9426521; doi:10.1128/mbio.01456-22)
Supplement: FIG S3 [file mbio.01456-22-sf003.pdf]

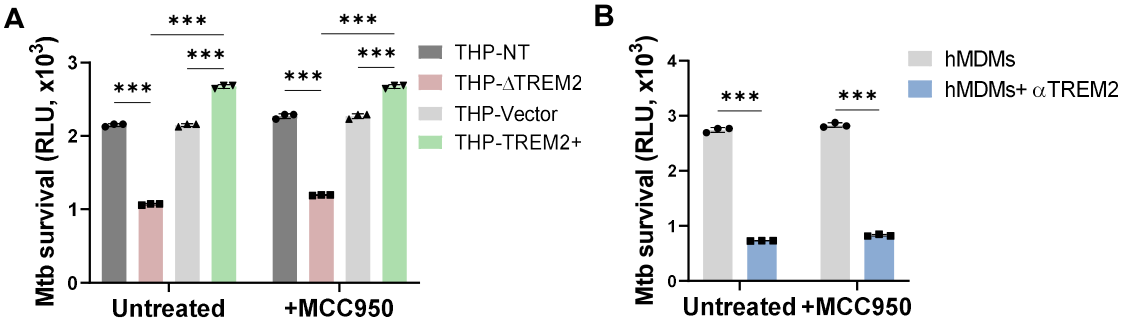

Figure S3. Enhanced bacterial clearance in TREM2 deficient or neutralized macrophages does not depend on NLRP3 activation. (A) THP-NT, THP-ΔTREM2, THP-Vector, and THP-TREM2+ macrophages, or **(B)** hMDMs with or without anti-TREM2 treatment were mock treated or pre-treated with 0.3 µM MCC950 for 24 h and infected with Mtb-lux (MOI = 10). *M. tuberculosis* viability (RLU) was analysed at day 4 post infection. Error bars in this figure represent the mean ± SD of three independent biological replicates.
