# Supplementary material for: TREM2 Promotes Immune Evasion by Mycobacterium tuberculosis in Human Macrophages
Source: mBio. 2022 Aug 4;13(4):e01456-22. doi: 10.1128/mbio.01456-22 (PMC9426521; doi:10.1128/mbio.01456-22)
Supplement: FIG S4 [file mbio.01456-22-sf004.pdf]

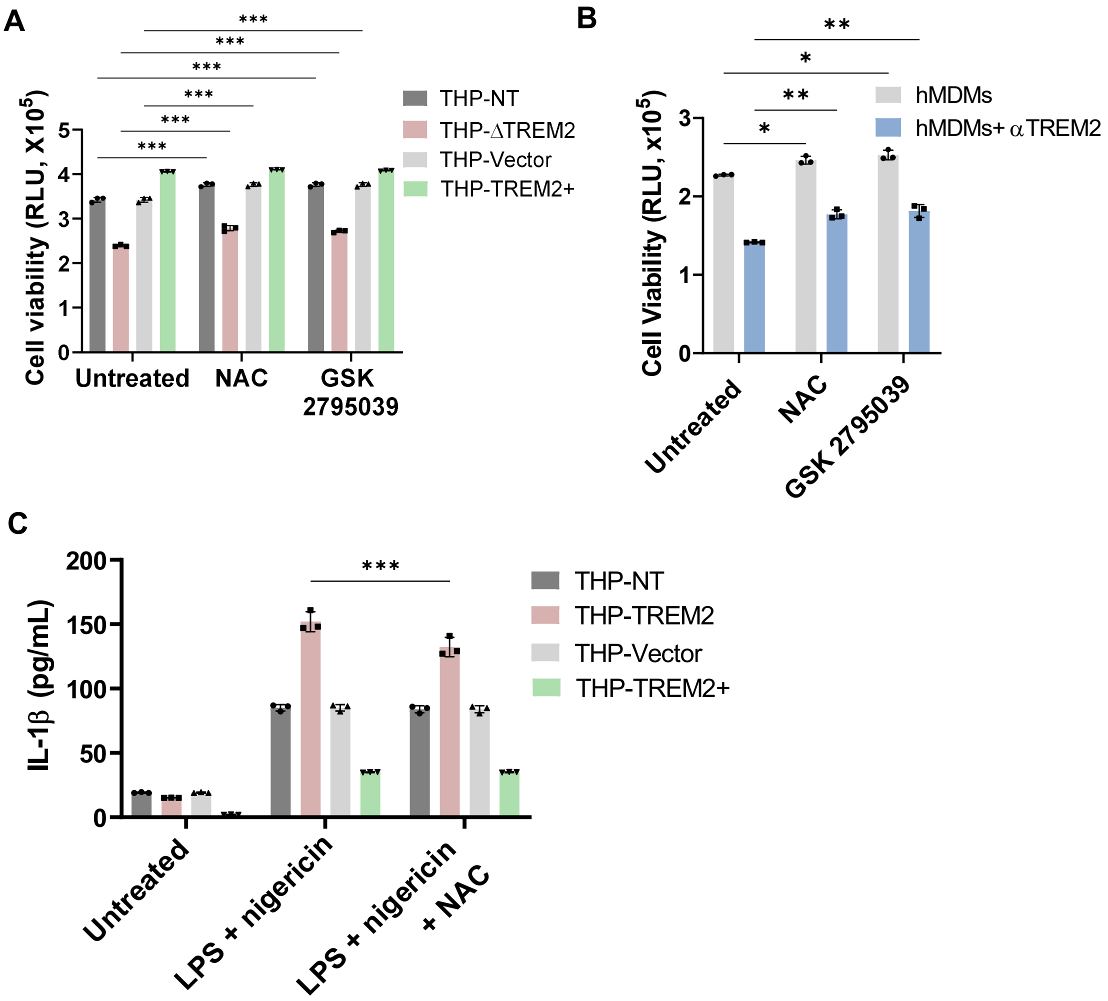

Figure S4. Inhibition of ROS partially restores cell viability in TREM2 deficient or neutralized macrophages during Mtb infection. (A) THP-1 macrophages (THP-NT, THP-ΔTREM2, and THP-TREM2+) and **(B)** hMDMs mock- or pre-treated with anti-TREM2, then treated with N-acetyl cysteine (NAC) or GSK2795039 for 24 h, followed by infection with Mtb. Macrophage viability was assessed at day 4 post-infection using CellTiter-Glo. **(C)** THP-NT, THP-ΔTREM2, and THP-TREM2+ macrophages were mock- or pre-treated with NAC for 24 h. Cells were then primed with LPS for 4 h prior to treatment with 5 μM nigericin for 24 h. IL-1β was measured by ELISA. Error bars in this figure represent the mean ± SD of three independent biological replicates.
